# Kinship and reproductive condition correlate with affiliation patterns in female southern Australian bottlenose dolphins

**DOI:** 10.1038/s41598-020-58800-2

**Published:** 2020-02-05

**Authors:** Fernando Diaz-Aguirre, Guido J. Parra, Cecilia Passadore, Luciana Möller

**Affiliations:** 1 Cetacean Ecology, Behaviour and Evolution Lab, College of Science and Engineering, Flinders University, South Australia, Australia; 2 Molecular Ecology Lab, College of Science and Engineering, Flinders University, South Australia, Australia

**Keywords:** Behavioural ecology, Social evolution

## Abstract

Social relationships in female mammals are usually determined by an interplay among genetic, endogenous, social and ecological factors that ultimately affect their lifetime reproductive success. However, few studies have attempted to control for, and integrate these factors, hampering our understanding of drivers underlying female sociality. Here, we used generalized affiliation indices, combined with social networks, reproductive condition, and genetic data to investigate drivers of associations in female southern Australian bottlenose dolphins. Our analysis is based on photo-identification and genetic data collected through systematic boat surveys over a two-year study period. Female dolphins formed preferred associations and social clusters which ranged from overlapping to discrete home ranges. Furthermore, matrilineal kinship and biparental relatedness, as well as reproductive condition, correlated with the strength of female affiliations. In addition, relatedness for both genetic markers was also higher within than between social clusters. The predictability of resources in their embayment environment, and the availability of same-sex relatives in the population, may have favoured the formation of social bonds between genetically related females and those in similar reproductive condition. This study highlights the importance of genetic, endogenous, social and ecological factors in determining female sociality in coastal dolphins.

## Introduction

Female reproductive success in mammals is limited by the costs of lactation, gestation and caring for their young^1^, which in turn places constraints on their distribution and behaviour^2,3^. Female social relationships and their spatial distribution are therefore largely determined by ecological factors affecting the quantity and quality of food they can obtain, which together with predation risk, influences the chances of offspring survival^4–6^. In most mammals, females tend to remain in their natal areas and associate in groups, but in some species they are known to leave their natal ranges or social groups to avoid local competition for resources and mating opportunities^7,8^. Females living in groups may benefit from reduced risk of predation, assistance in infant rearing, increased access to food resources, increased reproductive output, survival and psychological wellbeing, as well as protection from sexual coercion by males^4,8–13^. For example, enhanced offspring survival has been demonstrated in female yellow baboons (*Papio cynocephalus*) that show close social bonds^14,15^. Similarly, social factors have been attributed to partially drive calving success in bottlenose dolphins (*Tursiops* cf. *aduncus*^11^), and assistance in protection from male coercion^13,16^.

If social relationships have a positive effect on fitness (e.g.^11,14,15^), kin selection theory predicts that social bonds should preferentially form among relatives^17^. In agreement to this, kinship has been demonstrated to be an important factor on the development and maintenance of social bonds in many female mammals (but see^18^), including African elephants (*Loxodonta africana*^19^), sperm whales (*Physeter macrocephalus*^20^), giraffes (*Giraffa camelopardalis*^21^), spotted hyenas (*Crocuta crocuta*^22^) and rhesus macaques (*Macaca mulatta*^23,24^). Associating with kin can provide fitness benefits, such as those related to cooperative foraging, increased growth rates, enhanced reproductive success, reduced aggression, protection from predators, and shared social and ecological knowledge^11,14,25–29^. Moreover, the kin structure of a group has implications for the evolution of social behaviour^7^. When female groups are composed of close relatives, groups are usually stable and cooperation among females is common. In contrast, when groups are formed by non-related individuals, females usually move between social groups and cooperative behaviours are not as frequently observed (e.g.^30,31^). For example, the stability, quality and strength of social bonds in female yellow baboons correlate with maternal, and to a lesser extent, paternal relatedness between individuals^32,33^. Similarly, it has been shown that social cohesion in female yellow-bellied marmots (*Marmota flaviventris*) is maintained through affiliative interactions among related individuals^34^.

More recently, Cantor and Farine^35^ demonstrated, using agent-based models, that simple foraging interactions in competitive environments may give rise to the formation of stable social groups consisting of relatives. They showed that a process by which ‘individuals keep foraging with the same individuals if they were successful together in the past’ can lead to a stable social community structure and the emergence of kin-structure via fitness benefits or philopatry^35^. Their models, based on an energetic reward (food), provides an alternative evolutionary mechanism for the formation of stable, kin-based social groups similar to those observed in many vertebrates^35^.

Bottlenose dolphins (*Tursiops* spp.) live in societies with fission-fusion dynamics in which patterns of associations among individuals vary in strength and temporal stability^36^. Adult female bottlenose dolphins, apart from having a strong social bond with their calves for the first few years of the calf’s life, usually form loose to moderate associations with an extensive network of females of various ages and degrees of kinship^37–41^. In most well studied populations, females associate more closely within smaller clusters, called ‘bands’ or ‘cliques’. For example, in Sarasota Bay, USA, and Port Stephens, Australia, female common bottlenose dolphins (*T. truncatus*) and Indo-Pacific bottlenose dolphins (*T. aduncus*), respectively, form clusters or stable subsets of frequent associates that share similar core areas within their home ranges^37,39,42^.

As reported in other mammals, kinship appears to play an important role in shaping female associations in bottlenose dolphins. In Shark Bay (Western Australia) and Port Stephens (eastern Australia), female association patterns were positively correlated with genetic relatedness^39,40^, although at social cluster level, kinship was not a determinant of membership within social clusters^39^. Shared reproductive state also seems to play a role in delineating female associations in bottlenose dolphins^37,38,43^. Möller and Harcourt^43^ found that females in similar reproductive state had higher association levels than females in different states, probably related to similar energetic and protection requirements for females with dependent calves. In inshore habitats, where resources are likely to be more predictable^44^, Möller^45^ suggested that delphinid female philopatry may be favoured because of the benefits of familiarity with food resources. Furthermore, moderate social bonds may emerge between both kin and non-kin, although long-term social bonds may be more common between female kin^45^.

Here, we investigated the affiliation patterns and kinship relationships of female southern Australian bottlenose dolphins (*Tursiops* cf. *australis*^46^) inhabiting the inner area of Coffin Bay, South Australia, a heterogeneous inshore environment composed of small bays and channels. Coffin Bay is considered a stronghold for southern Australian bottlenose dolphins, with high densities of dolphins reported (1.57–1.70 dolphins/km^2^ ^47^), similar male-to-female ratio of non-calf individuals (males = 46–52; females = 52–60^47^), and restricted ranging patterns by both sexes^48^. The majority of females in Coffin Bay had representative ranges (95% kernel ranges) smaller than 15 km^2^ (mean representative range = 14.7, SD = 7.0 km^2^), and most (56%) showed ranging patterns restricted to a particular bay^48^. The Coffin Bay dolphin population is socially structured into two communities with discrete core ranges, where individuals from the same communities are on average more bi-parentally related than individuals from different communities^49^. Furthermore, males tend to form kin-based associations, which may enhance their access to females for mating^50^.

We used generalized affiliation indices (GAIs)^51^, and controlled for factors that could affect social analysis, such as home range overlap, gregariousness, and differences in the number of sightings among individuals^51,52^. Subsequently we combined these factors with social network techniques, and information on female’s reproductive condition and genetic relatedness, to investigate the factors driving associations in female southern Australian bottlenose dolphins. Based on predictions for female bonding in coastal delphinids^45^, we expect that females in Coffin Bay will exhibit preferred associates and form social groups, and these will likely be based on kinship relationships due to the availability of close relatives within the two dolphin communities identified in this population. In addition, given the presence of adult females in different reproductive status in the population (with and without dependent calves), similarity in their reproductive condition is expected to correlate with the strength of their associations.

## Results

A total of 152 boat surveys were conducted in Coffin Bay during the study period, with 967 dolphin groups sighted. A total of 657 groups were then selected based on criteria, which excluded identical groups resighted on the same day, and groups with less than 75% of dolphins identified. We included for the social analyses 55 females with more than 11 sightings, which were sighted within 550 groups. Of these females, 50 were identified by genetic sexing, and 5 based on the presence of a dependent calf on more than 10 separate days.

### Female affiliation patterns

We found a significant correlation between home range overlap, gregariousness and the association indices; therefore these structural variables were controlled for when estimating female GAIs. The number of sightings per female dyad did not show a significant correlation with the association indices, and therefore it was not controlled for during GAIs estimation (Table 1). GAIs using deviance residuals ranged from −4.52 to 7.06 (mean = −0.41; SD = 1.53; n = 1485). We detected the presence of non-random companionships among female dolphins using a permutation test (Observed SD = 0.1; Random SD = 0.08; p < 0.01), and using GAIs deviance residuals, 117 preferred, 1331 casual and 37 avoided female pairs were identified (Table 2).

**Table 1:** Effectiveness of predictor structural variables in explaining association indices between female southern Australian bottlenose dolphins (*Tursiops* cf. *australis*) in Coffin Bay, South Australia. Partial correlation coefficients and results of MRQAP tests were obtained using 10,000 permutations in SOCPROG 2.7 (Whitehead 2009).

Predictor variable	Partial correlation	MRQAP (p-value)
Home range overlap	0.43	<0.01
Gregariousness	0.16	<0.01
Sightings per dyad	0.03	0.06

**Table 2:** Mean GAIs and genetic relatedness, and number of pairs sharing haplotypes for each affiliation category of female southern Australian bottlenose dolphins (*Tursiops* cf. *australis*) identified in Coffin Bay, South Australia. Asterisks denotes mean genetic relatedness values that differed from random expectations.

Affiliation category	N of pairs	N of pairs with haplotype data	N of pairs with microsatellite data	Mean GAIs deviance (SD)	pairs sharing haplotype (%)	Mean genetic relatedness
Preferred	117	79	99	3.5 (0.79)	44 (55.7)	0.14*
Casual	1331	845	1054	−0.68 (0.99)	405 (48)	0.11
Avoided	37	22	23	−2.94 (0.45)	14 (63.7)	0.11

**Figure 1:**
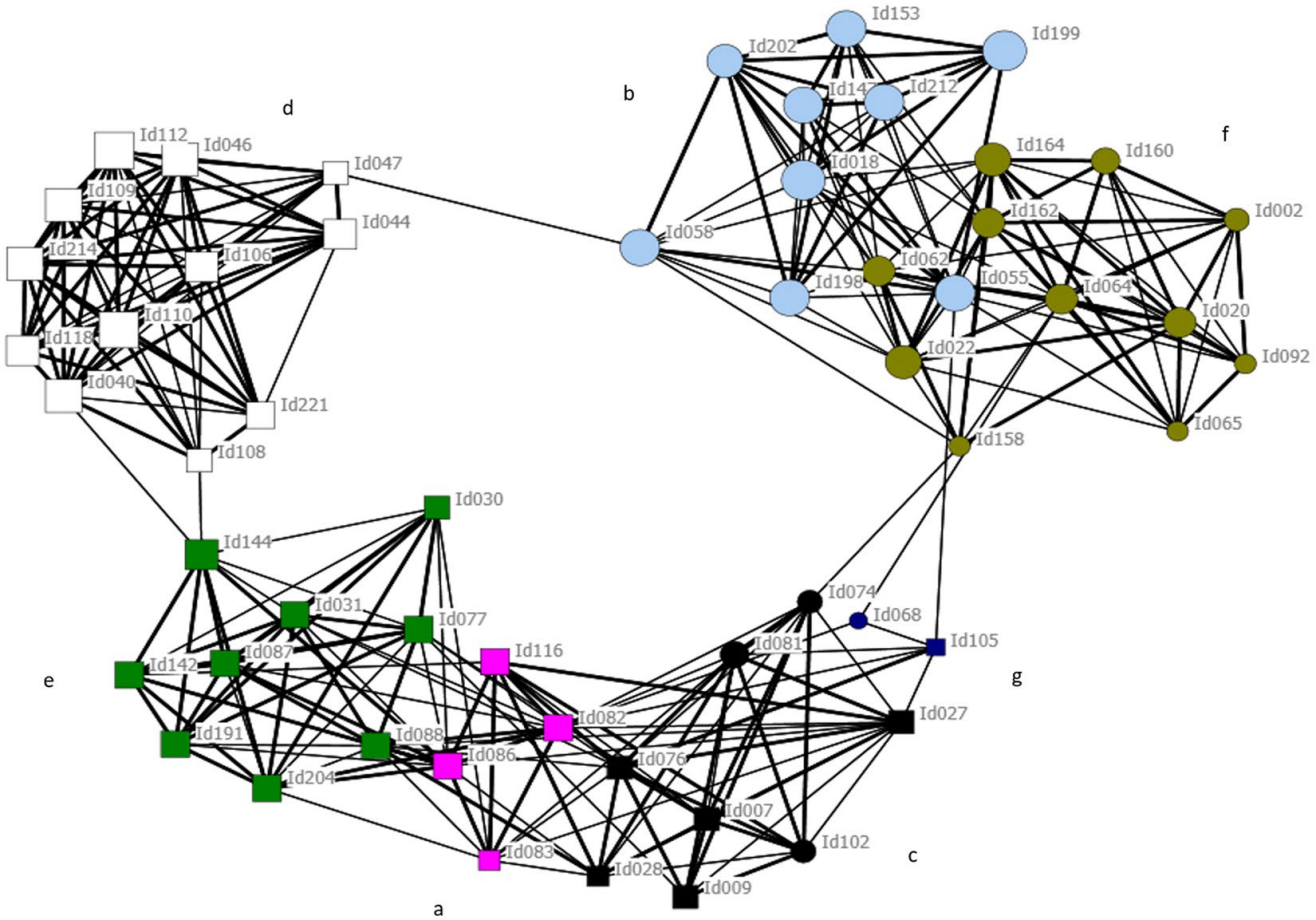
Social network of female southern Australian bottlenose dolphins (*Tursiops* cf. *australis*) in Coffin Bay, South Australia. The colour of the nodes represents the clusters identified using Newman’s modularity algorithm (denoted by a capital letter). Node sizes represent the gregariousness of the individuals while shape the communities identified at the population level: square and circles, Mt. Dutton-Kellidie Bay and Pt. Douglas communities, respectively^49^. Edge width is proportional to the affiliation index and only displayed for affiliation indices greater than 0.82 (twice the mean affiliation index over all female individuals).

**Figure 2:**
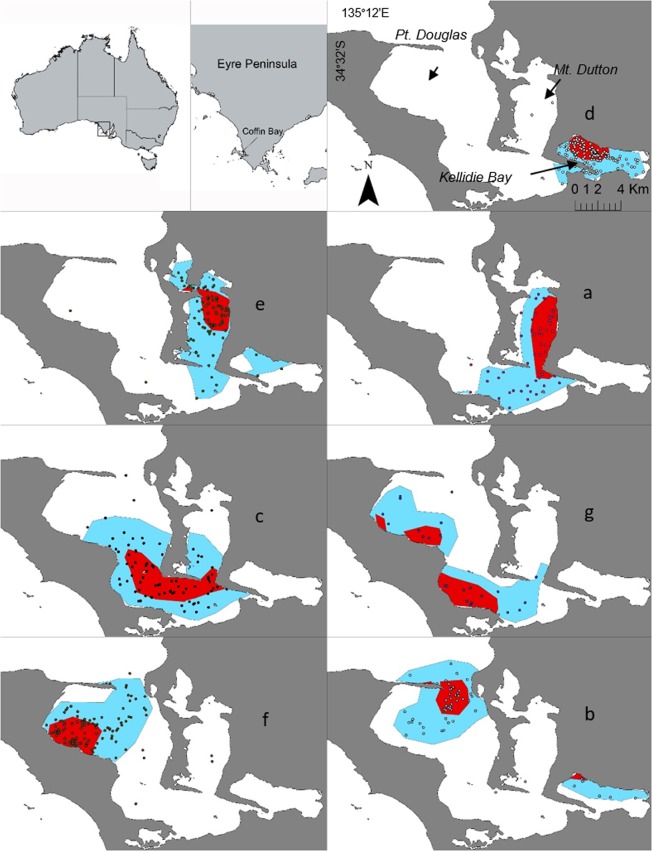
Map of Coffin Bay, South Australia, showing kernel density estimates (KDE) for each of the seven female social clusters of southern Australian bottlenose dolphins (*Tursiops* cf. *australis*). The red shades represent core areas (50% KDE) and blue shades are the representative ranges (95% KDE). Dots represent distinct groups of animals with colours and capital letters following those in the social network (Fig. 1). Maps were arranged according to the similarity in geographic distribution of each social group. For a more detailed analysis of female ranging patterns in this population see Passadore *et al*.^48^.

The Newman’s modularity clustering technique revealed that the Coffin Bay female population was subdivided into seven social clusters (Qmax = 0.45; Fig. 1; Table 3), which ranged in size from two to twelve individuals (mean = 7.9; SD = 3.63). Social clusters showed a mixture of ranging patterns (Fig. 2), with some clusters showing overlapping areas of usage and others using discrete areas in the Coffin Bay embayment. As anticipated, social clusters that were closer in the social network also showed similar areas of spatial use within the bay.

**Table 3:** Mean GAIs and genetic relatedness, and number of pairs sharing haplotypes for each of the seven female social clusters of southern Australian bottlenose dolphins (*Tursiops* cf. *australis*) identified in Coffin Bay, South Australia. Social cluster IDs are represented as in Fig. 1. Asterisks denotes mean genetic relatedness that differed from random expectations (p < 0.05).

Cluster ID	N of individuals (pairs)	Mean GAIs deviance (SD)	N pairs with haplotype data	N pairs with microsatellite data	Pairs sharing haplotype (%)	Mean genetic relatedness
A	4 (6)	3.68 (0.83)	3	3	1 (33.3)	0.02
B	9 (36)	2.18 (0.96)	10	10	9 (90)	0.23
C	8 (28)	2.58 (1.69)	28	28	13 (46)	0.09
D	12 (66)	2.6 (1.23)	55	66	25 (45.5)	0.14
E	9 (36)	2.8 (1.25)	15	36	10 (66.7)	0.13
F	11 (55)	1.92 (1.4)	36	45	27 (75)	0.14
G	2 (1)	1.55	1	1	0 (0)	0.13
Within clusters		2.5 (0.69)	148	189	85 (57.4)	0.14*
Between clusters		−0.8 (0.25)	798	987	369 (46.2)	0.11
All females		−0.41 (0.17)	946	1176	454 (48)	0.11

**Figure 3:**
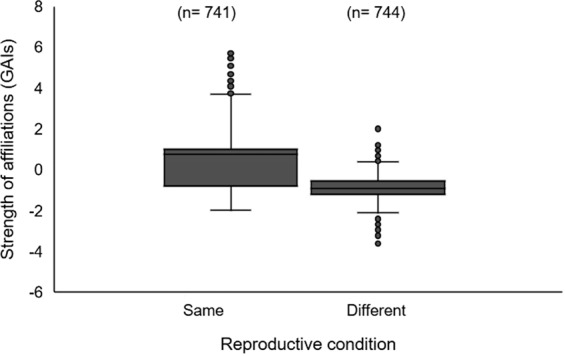
Box plot showing the strength of affiliations (GAIs values) with respect to the reproductive condition of female southern Australian bottlenose dolphins (*Tursiops* cf. *australis*) in Coffin Bay, South Australia. The middle line shows the median value, the rectangle indicates the first to the third quartiles, and the whiskers above and below the box, the minimum and maximum values, respectively. Circles above or below the whiskers represent outlier points.

We found a positive correlation between female reproductive condition and affiliation indices (*r* = 0.08; p < 0.05), and higher values of affiliation among females in similar reproductive state (Fig. 3; same reproductive state median GAIs = 0.76; different reproductive state median GAIs = −0.91; p < 0.001). Pairs of females in similar reproductive condition associated more often than those in different states; thus, females with calves associated more often with other females with calves, and females without calves associated more often with other females without calves.

### Affiliation patterns and kinship

**Figure 4:**
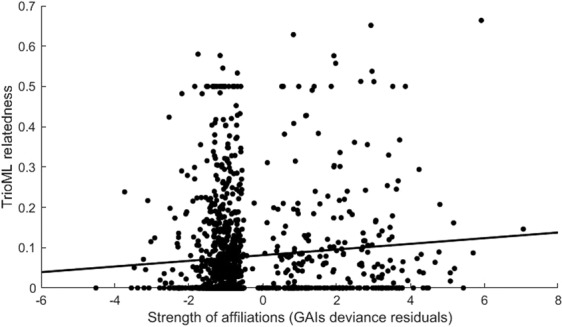
Relationship between affiliation indices and the triadic likelihood estimator (TrioML) of genetic relatedness in pairs of female southern Australian bottlenose dolphins (*Tursiops* cf. *australis*) inhabiting Coffin Bay, South Australia.

MtDNA control region haplotype and microsatellite data were gathered for 44 and 49 female dolphins considered in the social analysis, respectively. We only found two haplotypes, both with similar frequencies (A = 23; B = 21) in the female population. There was a significant correlation between the affiliation indices and both pairwise mtDNA haplotype sharing (*r* = 0.1; p < 0.05) and genetic relatedness based on microsatellites (*r* = 0.1; p < 0.05; Fig. 4).

Comparing different affiliation classes (preferred, casual and avoided), we found that preferred female affiliates had higher mean pairwise genetic relatedness than casual and avoided pairs (p < 0.05; Table 2). However, we did not detect a significant difference in the frequency of shared haplotypes for the different affiliation classes (p = 0.16; Table 2).

**Figure 5:**
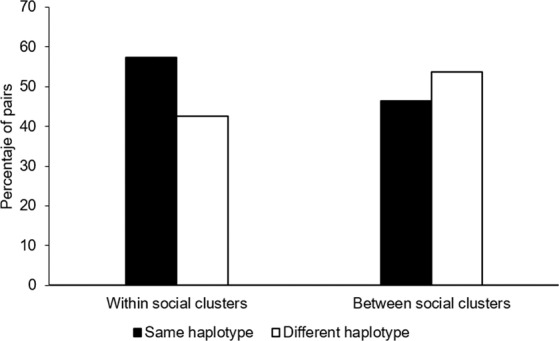
Proportion of mtDNA sharing among female southern Australian bottlenose dolphins (*Tursiops* cf. *australis*) within and between social clusters identified in Coffin Bay, South Australia.

At social cluster level, we found a higher frequency of mtDNA sharing (Fig. 5; Table 3; p < 0.05) and higher mean genetic relatedness (Table 3; p < 0.05) within than between social clusters. We did not find a significant correlation between genetic relatedness (r = 0.02; p = 0.8) or mtDNA haplotype sharing (r = 0.02; p = 0.3) and the home range overlap of the individuals.

## Discussion

We showed that female southern Australian bottlenose dolphins in Coffin Bay form moderate to strong affiliations and social clusters in which kinship generally correlated positively with their associations. Furthermore, we found that the reproductive condition of females correlated with the strength of affiliations. The patterns observed conforms to theoretical expectations for the formation of social bonds in female dolphins inhabiting inshore environments^45^, suggesting that similar evolutionary forces may be shaping the social behaviour of dolphin populations from disparate geographic areas and different species.

The formation of non-random associations in female mammals is known for several species, such as Asian elephants (*Elephas maximus*), giraffes^21^, spotted hyenas^53^, grey kangaroos, (*Macropus giganteus*^54^), zebras (*Equus grevyi*) and onagers (*Equus hemionus*^55^). In Coffin Bay, female dolphins form preferred associations and social clusters that are similar to those described in some other coastal populations of bottlenose dolphins (e.g.^36,37,39^). For example, in the Port Stephens’ dolphin population in eastern Australia, females generally associated at low to moderate levels with each other, but more closely with certain females forming social clusters^39^. Moreover, these social clusters used different spatial areas within the embayment, which were characterised by different habitats^39,56^. Female dolphins in our study area showed a similar pattern, establishing non-random associations with certain individuals and forming social clusters that showed spatial preferences for particular sub-embayments of Coffin Bay. The social and spatial divisions observed corresponded well with those reported at the population level in Coffin Bay, however, in clusters C and G some females were assigned as belonging to different communities^49^. This could be potentially explained by these two clusters inhabiting the home range area of overlap between the two communities detected at population level, probably acting as connectors between the communities (e.g.^57^). The formation of social clusters among females also support the idea of a hierarchically organized dolphin society in Coffin Bay^49^, similar to the one reported in Port Stephens^56^.

The formation of social groups in female bottlenose dolphins has been attributed to benefits leading to increased infant survival, defence against predators and male coercion, and lifetime fitness^11,13,36,39,43^. In Coffin Bay, although apparently low (Diaz-Aguirre, pers. obs.), the risk of predation could be influencing female behaviour (e.g.^58^). Great white sharks (*Carcharodon carcharias*) are relatively common in South Australian waters, and have been observed within our study area, with at least one dolphin observed with severe shark injuries likely inflicted by this species (Diaz-Aguirre, pers. obs.). In addition, male dolphins in Coffin Bay form small groups, likely alliances, which could function to increase their access to females^50^. Thus females may also benefit by forming social groups to defend against sequestering or coercion attempts by males (e.g.^13^).

In societies with fission-fusion dynamics, such as those of bottlenose dolphins (e.g.^36^), females associations may vary according to their developmental stage and reproductive condition, which in turn is affected by ecological and social pressures (e.g.^59,60^). In our study area, we observed that females in similar reproductive condition associated more often than those in different states. GAIs were higher between individuals in similar reproductive condition, and we also found a weak but significant correlation between the reproductive state and the strength of the affiliations. Our results are similar to those reported by Möller and Harcourt^43^, where association indices are higher among females in similar reproductive state. However, dissimilar methodologies for estimating associations between the studies prevent direct comparisons. Both studies, however, did not have the age class of the calves, so it is possible that females with calves prefer to associate with others with calves in similar developmental stage. It has been suggested that female bottlenose dolphins in similar reproductive condition may benefit by associating with each other because of similar energetic and protection requirements^37,43^. In Coffin Bay, females with calves may have different food requirements (e.g.^61^), and be under higher pressure of predation by sharks and injuries inflicted by coercing males (e.g.^62,63^), thus favouring associations among individuals in similar reproductive condition. We suggest that reproductive condition is a an important determinant of associations in our study area, however others factors such as philopatry, calf and/or mother age, social familiarity, maternal experience and kinship might also play a role in female sociality^43^.

The effect of philopatry, as home range overlap, was controlled during GAIs estimation. Despite the lack of age information for the females under study, our protocol for identifying individuals in the field, where all females included in the analysis were categorized as adults based on size, let us assume that most of the individuals were adults or about to reach sexual maturity. Thus, the potential for immature and adult animals associating together and confounding our results are unlikely. Due to the long-term nature of bottlenose dolphin associations^36^, it is possible that previously close associations may not dissipate completely if two females display different reproductive conditions^43^. Thus, some females close associates may include individuals with whom they previously shared similar reproductive states in the past^43^. Similarly, it has been shown that experienced mothers associate more often with females with calves when compared with primiparous mothers, which tend to associate with nulliparous females, males or females without calves^43,64^. Long-term photo-identification data of this population and comparisons of survival rates among female offspring inhabiting different areas of Coffin Bay, as well as observations of female-male interactions, could provide important information to further test hypotheses about the role of reproductive state on female group associations.

The importance of kinship in the establishment and maintenance of social relationships has been documented in a wide variety of mammals (e.g. lions, *Panthera leo*^65^, elephants^19^, hyenas^22^). Moreover, the establishment of social bonds among philopatric female kin has been suggested as one of the initial steps in the evolution of mammal sociality^66^. Similarly, a recent modelling study has suggested that simple specialization for resources among competing groups might promote the formation of stable social groups in which kin structure may develop via fitness benefits or philopatry^35^. In this study, we have demonstrated that association patterns in female southern Australian bottlenose dolphins are correlated positively with both matrilineal and bi-parental relatedness. In addition, we found higher levels of relatedness and haplotype sharing within than between female social clusters. However, when considering different affiliation classes, we only found support for higher bi-parental relatedness among preferred female pairs, not haplotype sharing. This last result may be partly affected by the low-explanatory power of the maternally inherited marker as only two haplotypes where found in the population. Moreover, the lack of correlation between home range overlap and genetic relatedness suggests that the associations are likely driven by kinship, and do not represent a by-product of the correlation between these two variables. This also suggests that some females could be dispersing from their natal areas into adjacent bays, which differs from the general pattern observed in inshore bottlenose dolphins, where females usually remain philopatric (i.e.^36,44,45,67^). Under this scenario, kin clusters of preferred associates may relocate to different areas or embayment within Coffin Bay in response to social or ecological pressures, providing an explanation for the lack of correlation between home range overlap and genetic relatedness.

We propose that kinship, likely driven by fitness benefits, plays an important role in the formation of social preferences among female dolphins in Coffin Bay. A positive correlation between association patterns and kinship has been previously reported in cetaceans (e.g. sperm whales^20^) and in other bottlenose dolphins populations inhabiting inshore environments (e.g.^39,40^). In a review of delphinid socio-genetic structure, Möller^45^ proposed that long-term social bonds in female dolphins occurred mainly among kin, while moderate social bonds may emerge between related and non-related individuals. Thus, the associations among non-related individuals observed in our study may reflect short-term preferences on a larger temporal scale given the long-lived nature of these animals. This could be attributed to ecological factors, such as fluctuations in prey resources, which may promote the emigration of individuals into other non-related social groups when competition is high within their core social clusters. Long-term data on the association patterns of these dolphins may provide important information to better understand the interactions between kinship, philopatry and association patterns.

Our results corroborate predictions from previous studies that in inshore habitats where resources are relatively predictable^44^, female dolphins may benefit by forming social bonds with kin and other females in similar reproductive condition, while maintaining moderate and loose social bonds with some same sex individuals^41,43,45^. The inner area of Coffin Bay is a complex inshore environment, where females exhibit a high degree of site fidelity, and range over relatively small areas, likely due to predictable food resources within the small sub-embayments^48^. Our results also provide insights into the mechanism promoting the formation of male associations based on kinship relationships in Coffin Bay^50^. Considering that the population is relatively large^47^ and organised into two social communities^49^, and that females prefer to associate with kin and with others in similar reproductive condition, young male calves may encounter opportunities to associate and develop social bonds with other males that are genetically related. As males grow older these associations may become stronger and form the basis for the formation of social preferences observed in the adult male population^50^.

In summary, our findings demonstrate that kinship and reproductive condition are important factors influencing association patterns of female southern Australian bottlenose dolphins in Coffin Bay, South Australia. Long-term behavioural observations for estimating calving success and how this relates with female age, maternal experience, social network metrics, kinship relationships and areas of usage would provide important information into the functional mechanisms promoting long-lasting female associations and the formation of social clusters. Our results add to the growing knowledge which demonstrates the interplay among social, genetic, endogenous and ecological factors shaping dolphins and other complex mammalian societies.

## Methods

### Ethics statement/approval

This study was carried out under Flinders University Animal Welfare Committee approval number E310 and under permits to undertake scientific research: E26171-1, E26171-2, E26171-3 and MR00056-1 from the Department of Environment, Water and Natural Resources (DEWNR), South Australia, and under S115 ministerial exemptions (MEs: 9902601, 9902660, 9902714 and 9902779) from Primary Industries Resources South Australia (PIRSA)^49^. Researchers in the field attempted to minimize disturbance to dolphin groups by following the Australian guidelines for whale and dolphin watching^68^. Briefly, dolphins were approached using a constant speed and following a parallel movement to their direction of travel. For obtaining biopsy samples from animals, we used two methods that produce minimum short- and long-term impacts on dolphins following the guidelines reported in^69^ and^70^. In addition, biopsy samples were only obtained from adult animals.

### Study area and data collection

Periodic boat surveys were conducted in Coffin Bay’s inner area (123 Km^2^), South Australia, between March 2013 and October 2015 (Fig. 2). The study area is characterised as a shallow reverse estuary (depths ranging from 1 to 12 meters) and is composed of three different bays that differ in depth and habitat types. Mt. Dutton and Kellidie Bay are mainly dominated by shallow seagrass habitats, whereas Pt. Douglas is deeper and contains a diverse array of habitats, including tidal sandflats, temperate reefs, seagrass meadows and deeper waters with sandy bottoms^71,72^. Boat surveys were conducted in calm sea conditions (Beaufort < 3) and were designed to cover all different habitat types and seasons within the study area^47^. More information on survey design and transects followed during the study are reported in^47^.

Dolphin groups sighted were approached to record their location, composition and size, and to obtain photographs of their dorsal fins using DSLR cameras equipped with 100–300 mm and 100–400 mm zoom lenses. Individual dolphins were characterized using natural marks on their dorsal fins^73^ and the best images of each individual in a group were classified in Discovery v1.2^74^. A detailed description of the protocols used for classifying photographs can be found in^47^. We classified individuals >1.5 m in length as non-calves and those ≤1.5 m and closely accompanied by a non-calf individual as calves^50^. In addition, we collected biopsy samples from photo-identified non-calf individuals using the biopsy pole system for bow-riding dolphins^69^, or the PAXARMS remote biopsy system for dolphins^70^. Samples were stored in a 20% DMSO salt saturated solution and frozen at −20 °C following recommendations^75^.

### Genetic analyses

DNA extraction was performed using proteinase K digestion followed by a salting-out protocol^50,76^. Extracted DNA was amplified at 11 microsatellite loci following the conditions reported in^77^. Samples were genotyped on an Applied Biosystems 3130 DNA Analyser and allele size fragments were scored using GENEMAPPER v4.1 (Applied Biosystems). The software MICROCHECKER^78^ was used to test for the potential presence of null alleles and allelic dropout. GENEPOP v4.2^79^ was used to assess Hardy-Weinberg equilibrium (HWE) and linkage disequilibrium using 1,000 iterations. There is no evidence of genetic subdivision within the inner area of Coffin Bay for the southern Australian bottlenose dolphin population^50,77^.

We amplified a region of 450 base pairs (bp) of the mitochondrial DNA (mtDNA) control region using primers Dlp-5 (5′-CCA TCG WGA TGT CTT ATT TAA GRG GAA-3′) and Dlp-1.5 (5′-TCA CCC AAA GCT GRA RTT CTA-3′)^80^, following conditions detailed in^81^. PCR products were sequenced on an Applied Biosystems 3130xl genetic analyser. SEQUENCHER v5.2.4 (Gene Codes Corporation, Ann Arbor, MI, USA) was used to align the sequences resulting in a high-quality 437 bp fragment. To genetically determine the sex of each biopsied dolphin, we amplified a fragment of the ZFX and SRY genes using the protocols described in^82^.

### Defining female associations and estimating generalized affiliation indices

In this study we considered a group of dolphins as all individuals within a 100 m radius and participating in similar behavioural activities^37,50^. For subsequent analyses we only considered those groups in which a minimum of 75% of the individuals were photo-identified based on the estimated group size for each sighting^50^. We only included the first sighting of an individual in each survey day, and subsequent sightings of identical groups in the same day were excluded. All dolphins identified in the same group were considered associated, and if new individuals joined a group during a sighting these were included in the same group^50^. To reduce the potential for false null associations among individuals with low number of sightings, we only considered dolphins observed on more than 11 occasions (equals to the median number of sightings for the population)^50^. In addition, we controlled for the cumulative number of sightings of each given dolphin pair during the affiliation index estimation (see below)^50^. In the subsequent analyses, we only included non-calf individuals positively identified as females either through the molecular sexing analysis, or by visual observation of a closely associated calf on more than 10 separate days.

We used generalized affiliation indices (GAIs^51^) to estimate the strength of associations between pairs of females. Using this method, we corrected for the correlation between association indices and structural variables known to affect social analyses (e.g.^51^). Using the half-weight index (HWI^83^), we constructed a matrix of associations and tested the above using controlled multiple regression quadratic assignment procedures (MRQAP): cumulative number of sightings for each dolphin pair, gregariousness (number of associates of an individual^84^), and home range overlap^50^. Variables that showed a significant correlation with the HWI were retained and used for GAIs estimation. The controlled generalized affiliation indices were used for subsequent social analyses. We did not include reproductive condition or genetic relatedness as structural variables within the GAIs because we were interested in evaluating the influence of these variables on the strength of the affiliations (as suggested by Whitehead^51^).

We used the package AdehabitatHR^85^ in R v3.2.3^86^ to estimate individual dolphins 95% utilization distributions. We used the href function to estimate the smoothing parameter (h) and subsequently visually explored individual ranges to determine the value that best adjusted to the data set. A value of h = 550 was chosen as this provided the best representation for the data^50^. Home range overlap between dolphin pairs was subsequently estimated using the kernel-based utilization distribution overlap index method^87^ in AdehabitatHR^85^. SOCPROG 2.7^88^ was used to calculate gregariousness, to carry MRQAP and to estimate GAIs.

### Analysis of female affiliation patterns

We used two different approaches for testing preferred and avoided companionships among female dolphins in Coffin Bay. First we used the permutation method implemented by Bejder *et al*.^89^ and modified by Whitehead^88^, using daily sampling periods to avoid demographic effects^90^. For this procedure we randomly permuted the affiliation matrices until P values stabilized using the standard deviations of the affiliation indices as test statistic^50^. In addition, we converted GAIs raw residuals into deviance residuals for identifying pairs of dolphins that demonstrated preferred, casual or avoided affiliations. These was done following recommendations by Whitehead and James^51^, who considered pairs with values above 2.5 as preferred, between 2.5 and −2.5 as casual, and below −2.5 as avoided companionships^50^.

Furthermore, we tested if females in similar reproductive condition associated more often than those in different states. We constructed a similarity matrix for females according to two categories: (1) observed with a dependent calf for >18 months and (2) observed without a calf or observed with a calf for <6 months. The time frame selected allowed us to differentiate females that were observed with calves during most of the study period from those that did not have calves or had them but for only a short period of time, either because the calf died or was weaned soon after the start of the study. Thus, (i) two females in category 1 (or two in category (2) were assigned a similarity value of 1; and (ii) two females, one in category 1 and the other in category 2 were assigned a similarity value of 0. In category 1 we included 24 females that were observed accompanied by a calf for at least 18 months. In category 2 we included two females that had calves for 4 and 5 months, and 29 females that were never observed with a calf. We tested for a correlation between the similarity matrix and the affiliation indices using a Mantel test with 10,000 permutations in SOCPROG 2.7^88^. In addition, we tested for differences in the affiliation index values with respect to the females’ reproductive condition (same or different) using a Mann-Whitney test.

To examine potential social divisions among female dolphins in Coffin Bay, we used Newman’s modularity matrix clustering technique^91–93^ implemented in SOCPROG 2.7^88^. This technique attempts to divide the population into social modules that have higher affiliation indices between members of the same social group than expected by chance using an eigenvector-based method^92,93^. Values of modularity above 0.3 are considered to provide a good description of the data^92^. Social network diagrams were subsequently constructed using the spring-embedded method implemented in NETDRAW v2.1.5.5^94^ to display female social groups and affiliations. In addition, to characterize the spatial distribution of the social clusters identified, we allocated the positions of the group sightings to the different social modules, and calculated representative (95%) and core (50%) ranges using the utilization distribution method implemented in AdehabitatHR^85^.

### Kinship relationships and genetic relatedness

To assess the role that kinship plays on female affiliations, we evaluated the degree of genetic relatedness at three different levels of associations. First, we tested for a correlation between bi-parentally and maternally inherited DNA markers and the strength of the affiliations (GAIs). We constructed a matrix of pairwise haplotype sharing for the mtDNA dataset and a matrix of pairwise genetic relatedness for the microsatellite dataset. Individuals with identical or different haplotypes were assigned a value of 1 or 0, respectively. Pairwise microsatellite genetic relatedness was estimated using the triadic likelihood estimator (TrioML^95^) in COANCESTRY v 1.0.1.5^96^. To evaluate the correlation between the strength of affiliations (GAIs) and both matrices, we used Mantel tests (with 10,000 permutations) in SOCPROG 2.7^50,88^.

Second, we compared the frequencies of shared haplotypes among preferred, casual and avoided affiliates using a randomization chi-square test with 10,000 Monte Carlo permutations. In addition, mean pairwise genetic relatedness was compared to random expectations among preferred, casual and avoided affiliates using 10,000 permutations in PERM^97^.

Third, we investigated whether females identified in the same social cluster had higher mean pairwise relatedness or higher frequencies of shared haplotypes than those of different clusters. We compared mean pairwise genetic relatedness and frequencies of haplotype sharing within and between social clusters using 10,000 permutations in COANCESTRY v1.0.1.5^96^, and a randomization chi-square test with 10,000 permutations, respectively.

Finally, to discard the possible association between genetic relatedness and philopatry, which could confound our kinship analysis, we tested for a correlation between the individuals’ home range overlap and their genetic relatedness (maternal and bi-parental separately) using Mantel tests with 10,000 permutations in SOCPROG 2.7^88^.

## Data Availability

Data made available to all interested researchers upon reasonable request to Fernando Diaz-Aguirre (fernandobiologist@gmail.com).

## References

[1] Clutton-Brock TH, Albon SD, Guinness FE (1989). Fitness costs of gestation and lactation in wild mammals. Nature.

[2] Whitehead H (1996). Babysitting, dive synchrony, and indications of alloparental care in sperm whales. Behav. Ecol. Sociobiol..

[3] Whitehead, H. *S*perm whales: social evolution in the ocean. (University of Chicago Press, 2003).

[4] Wrangham RW (1980). An Ecological Model of Female-Bonded Primate Groups. Behaviour.

[5] Clutton-Brock TH (1989). Review Lecture: Mammalian Mating Systems. Proc. R. Soc. B. Biol. Sci..

[6] Wittemyer G, Douglas-Hamilton I, Getz WM (2005). The socioecology of elephants: analysis of the processes creating multitiered social structures. Anim. Behav..

[7] Clutton‐Brock TH, Lukas D (2012). The evolution of social philopatry and dispersal in female mammals. Mol. Ecol..

[8] Wrangham, R. W. & Rubenstein, D. I. Social evolution in birds and mammals. In *Ecological Aspects of Social Evolution: Birds and Mammals* (eds. Rubenstein, D. I. & Wrangham, R. W.) 452–470 (Princeton University Press, 1986).

[9] Smuts BB, Smuts RW (1993). Male-Aggression and Sexual Coercion of Females in Nonhuman-Primates and Other Mammals - Evidence and Theoretical Implications. Adv. Study Behav..

[10] Massen J, Sterck E, de Vos H (2010). Close social associations in animals and humans: functions and mechanisms of friendship. Behaviour.

[11] Frere CH (2010). Social and genetic interactions drive fitness variation in a free-living dolphin population. Proc. Natl. Acad. Sci..

[12] Kelley JL, Morrell LJ, Inskip C, Krause J, Croft DP (2011). Predation risk shapes social networks in fission-fusion populations. PLoS One.

[13] Wallen MM, Patterson EM, Krzyszczyk E, Mann J (2016). The ecological costs to females in a system with allied sexual coercion. Anim. Behav..

[14] Silk JB, Alberts SC, Altmann J (2003). Social bonds of female baboons enhance infant survival. Science.

[15] Silk JB (2009). The benefits of social capital: close social bonds among female baboons enhance offspring survival. Proceedings. Biol. Sci..

[16] Connor R, Mann J, Watson-Capps J (2006). A Sex-Specific Affiliative Contact Behavior in Indian Ocean Bottlenose Dolphins, Tursiops sp. Ethology.

[17] Hamilton WD (1964). The genetical evolution of social behaviour. J. Theor. Biol..

[18] Langergraber K, Mitani J, Vigilant L (2009). Kinship and social bonds in female chimpanzees (*Pan troglodytes*). Am. J. Primatol..

[19] Archie EA, Moss CJ, Alberts SC (2006). The ties that bind: genetic relatedness predicts the fission and fusion of social groups in wild African elephants. Proc. R. Soc. B Biol. Sci..

[20] Gero S, Engelhaupt D, Whitehead H (2008). Heterogeneous social associations within a sperm whale, Physeter macrocephalus, unit reflect pairwise relatedness. Behav. Ecol. Sociobiol..

[21] Carter KD, Seddon JM, Frère CH, Carter JK, Goldizen AW (2013). Fission-fusion dynamics in wild giraffes may be driven by kinship, spatial overlap and individual social preferences. Anim. Behav..

[22] Holekamp KE, Sakai ST, Lundrigan BL (2007). Social intelligence in the spotted hyena (Crocuta crocuta). Philos. Trans. R. Soc. Lond. B. Biol. Sci..

[23] Widdig A, Nürnberg P, Krawczak M, Streich WJ, Bercovitch FB (2001). Paternal relatedness and age proximity regulate social relationships among adult female rhesus macaques. Proc. Natl. Acad. Sci. USA.

[24] Widdig A, Nürnberg P, Krawczak M (2002). & Streich, Wolf JürgenBercovitch, F. Affiliation and aggression among adult female rhesus macaques: a genetic analysis of paternal cohorts. Behaviour.

[25] Olsén KH, Järvi T (1997). Effects of kinship on aggression and RNA content in juvenile Arctic charr. J. Fish Biol..

[26] Silk JB (2002). Kin selection in primate groups. International Journal of Primatology.

[27] Krützen M, Barré LM, Connor RC, Mann J, Sherwin WB (2004). ‘O father: Where art thou?’ - Paternity assessment in an open fission-fusion society of wild bottlenose dolphins (Tursiops sp.) in Shark Bay, Western Australia. Mol. Ecol..

[28] Gerlach G, Hodgins-Davis A, MacDonald B, Hannah RC (2007). Benefits of kin association: related and familiar zebrafish larvae (Danio rerio) show improved growth. Behav. Ecol. Sociobiol..

[29] Smith JE (2014). Hamilton’s legacy: Kinship, cooperation and social tolerance inmammalian groups. Anim. Behav..

[30] Sterck EHM (1997). Determinants of female dispersal in Thomas langurs. Am. J. Primatol..

[31] Clutton-Brock T (2009). Structure and function in mammalian societies. Philos. Trans. R. Soc. B Biol. Sci..

[32] Silk JB, Altmann J, Alberts SC (2006). Social relationships among adult female baboons (papio cynocephalus) I. Variation in the strength of social bonds. Behav. Ecol. Sociobiol..

[33] Silk JB, Alberts SC, Altmann J (2006). Social relationships among adult female baboons (Papio cynocephalus) II. Variation in the quality and stability of social bonds. Behav. Ecol. Sociobiol..

[34] Wey TW, Blumstein DT (2010). Social cohesion in yellow-bellied marmots is established through age and kin structuring. Anim. Behav..

[35] Cantor M, Farine DR (2018). Simple foraging rules in competitive environments can generate socially structured populations. Ecol. Evol..

[36] Connor, R., Wells, R., Mann, J. & Read, A. The bottlenose dolphin: social relationships in a fission-fusion society. in Cetacean societies: field studies of dolphins and whales (eds. Mann, J., Connor, R. C., Tyack, P. L. & Whitehead, H.) 91–126 (The University of Chicago Press, 2000).

[37] Wells, R. S., Scott, M. D. & Irvine, A. B. The social structure of free-ranging bottlenose dolphins. in *Current Mammalogy* (ed. Genoways, H. H.) 247–305, 10.1007/978-1-4757-9909-5_7(Springer US, 1987).

[38] Smolker RA, Richards AF, Connor RC, Pepper JW (1992). Sex Differences in Patterns of Association Among Indian Ocean Bottlenose Dolphins. Behaviour.

[39] Möller LM, Beheregaray LB, Allen SJ, Harcourt RG (2006). Association patterns and kinship in female Indo-Pacific bottlenose dolphins (*Tursiops aduncus*) of southeastern Australia. Behav. Ecol. Sociobiol..

[40] Frère CH (2010). Home range overlap, matrilineal and biparental kinship drive female associations in bottlenose dolphins. Anim. Behav..

[41] Wiszniewski J, Lusseau D, Möller LM (2010). Female bisexual kinship ties maintain social cohesion in a dolphin network. Anim. Behav..

[42] Möller, L. M. Social organization and genetic relationships of coastal bottlenose dolphins in southeastern Australia. (Macquaire, 2001).

[43] Möller LM, Harcourt RG (2008). Shared reproductive state enhances female associations in dolphins. Res. Lett. Ecol..

[44] Gowans S, Würsig B, Karczmarski L (2008). The social structure and strategies of delphinids: predictions based on an ecological framework. Adv. Mar. Biol..

[45] Möller LM (2012). Sociogenetic structure, kin associations and bonding in delphinids. Mol. Ecol..

[46] Charlton-Robb K (2011). A new dolphin species, the burrunan dolphin (*Tursiops australis sp. nov*.), endemic to Southern Australian coastal waters. PLoS One.

[47] Passadore C, Möller L, Diaz-Aguirre F, Parra GJ (2017). Demography of southern Australian bottlenose dolphins living in a protected inverse estuary. Aquat. Conserv. Mar. Freshw. Ecosyst..

[48] Passadore C, Möller L, Diaz-Aguirre F, Parra GJ (2018). High site fidelity and restricted ranging patterns in southern Australian bottlenose dolphins. Ecol. Evol..

[49] Diaz-Aguirre, F., Parra, G. J., Passadore, C. & Möller, L. Genetic relatedness delineates the social structure of southern Australian bottlenose dolphins. *Behav. Ecol*. (2019).

[50] Diaz-Aguirre, F., Parra, G. J., Passadore, C. & Möller, L. M. Kinship influences social bonds among male southern Australian bottlenose dolphins (Tursiops cf. australis). *Behav. Ecol. Sociobiol*, 10.1007/s00265-018-2621-4 (2018).

[51] Whitehead H, James R (2015). Generalized affiliation indices extract affiliations from social network data. Methods Ecol. Evol..

[52] Farine, D. R. & Strandburg-Peshkin, A. Estimating uncertainty and reliability of social network data using Bayesian inference. *R. Soc. Open Sci*. **2** (2015). 10.1098/rsos.150367 PMC4593693 26473059

[53] Smith JE, Memenis SK, Holekamp KE (2007). Rank-related partner choice in the fission–fusion society of the spotted hyena (Crocuta crocuta). Behav. Ecol. Sociobiol..

[54] Best EC, Dwyer RG, Seddon JM, Goldizen AW (2014). Associations are more strongly correlated with space use than kinship in female eastern grey kangaroos. Anim. Behav..

[55] Sundaresan SR, Fischhoff IR, Dushoff J, Rubenstein DI (2007). Network metrics reveal differences in social organization between two fission–fusion species, Grevy’s zebra and onager. Oecologia.

[56] Wiszniewski J, Allen SJ, Möller LM (2009). Social cohesion in a hierarchically structured embayment population of Indo-Pacific bottlenose dolphins. Anim. Behav..

[57] Lusseau D, Newman MEJ (2004). Identifying the role that animals play in their social networks. Proc. R. Soc. London. Ser. B Biol. Sci..

[58] Heithaus MR, Dill LM (2002). Food availability and tiger shark predation risk influence bottlenose dolphin habitat use. Ecology.

[59] Mann J, Smuts B (1999). Behavioral development in wild bottlenose dolphin newborns (Tursiops sp.). Behaviour.

[60] Gibson, Q. & Mann, J. *Early social development in wild bottlenose dolphins: Sex differences, individual variation and maternal influence*. *Animal Behaviour* **76** (2008).

[61] Bernard HJ, Hohn AA (1989). Differences in Feeding Habits between Pregnant and Lactating Spotted Dolphins (Stenella attenuata). J. Mammal..

[62] Corkeron PJ, Morris RJ, Bryden MM (1987). Interactions between bottlenose dolphins and sharks in Moreton Bay, Queensland. Aquat. Mamm..

[63] Mann J, Watson-Capps JJ (2005). Surviving at sea: ecological and behavioural predictors of calf mortality in Indian Ocean bottlenose dolphins, Tursiops sp. Anim. Behav..

[64] C, O. A comparison of maternal care by primiparous and multiparous bottlenose dolphins. Does parenting improve with experience? (University of California, Santa Cruz, USA., 2001).

[65] Packer C, Gilbert DA, Pusey AE, O’Brieni SJ (1991). A molecular genetic analysis of kinship and cooperation in African lions. Nature.

[66] Perrin N, Lehmann L (2001). Is sociality driven by the costs of dispersal or the benefits of philopatry? A role for kin-discrimination mechanisms. Am. Nat..

[67] Rendell, L., Cantor, M., Gero, S., Whitehead, H. & Mann, J. Causes and consequences of female centrality in cetacean societies. *Philosophical Transactions of the Royal Society B: Biological Sciences* **374** (2019). 10.1098/rstb.2018.0066 PMC6664132 31303160

[68] Australian National Guidelines for Whale and Dolphin Watching. *Department of energy and environment* (2017). Available at, http://www.environment.gov.au/marine/publications/australian-national-guidelines-whale-and-dolphin-watching-2017.

[69] Bilgmann K, Griffiths OJ, Allen SJ, Möller LM (2007). A biopsy pole system for bow-riding dolphins: Sampling success, behavioral responses, and test for sampling bias. Mar. Mammal Sci..

[70] Krützen M (2002). A biopsy system for small cetaceans: Darting success and wound healing in Tursiops SPP. Mar. Mammal Sci..

[71] Saunders, B. *Shores and shallows of Coffin Bay*. *An identification guide*. (Australian Printing Specialists, 2012).

[72] Kämpf, J. & Ellis, H. Hydrodynamics and flushing of Coffin Bay, South Australia: A small tidal inverse estuary of interconnected bays. *J. Coast. Res*. 447–456, 10.2112/JCOASTRES-D-14-00046.1 (2014).

[73] Würsig, B. & Jefferson, T. A. *Methods of photo-identification for small cetaceans*. *Individual recognition of cetaceans: Use of photo identification and other techniques to estimate population parameters* (International Whaling Commission, 1990).

[74] Gailey, G. & Karczmarski, L. Discovery: photo-identification data-management system for individually recognizable animals (2013).

[75] Amos, B. & Hoelzel, A. Long-term preservation of whale skin for DNA analysis. Genetic ecology of whales and dolphins. *Rep. Int. Whal. Commision*, 99–103 (1991).

[76] Sunnucks P, Hales DF (1996). Numerous transposed sequences of mitochondrial cytochrome oxidase I-II in aphids of the genus Sitobion (Hemiptera: Aphididae). Mol. Biol. Evol..

[77] Pratt EAL (2018). Hierarchical metapopulation structure in a highly mobile marine predator: the southern Australian coastal bottlenose dolphin (Tursiops cf. australis). Conserv. Genet..

[78] Van Oosterhout C, Hutchinson WF, Wills DPM, Shipley P (2004). MICRO-CHECKER: Software for identifying and correcting genotyping errors in microsatellite data. Mol. Ecol. Notes.

[79] Raymond M, Rousset F (1995). GENEPOP (Version 1.2): Population Genetics Software for Exact Tests and Ecumenicism. J. Hered..

[80] Baker CS (1993). Abundant mitochondrial DNA variation and world-wide population structure in humpback whales. Proc. Natl. Acad. Sci. USA.

[81] Möller LM, Beheregaray LB (2001). Coastal bottlenose dolphins from south-eastern Australia are Tursiops aduncus according to sequences of the mitochondrial DNA control region. Mar. Mammal Sci..

[82] Gilson A, Syvanen M, Levine K, Banks J (1998). Deer gender determination by polymerase chain reaction: validation study and application to tissues, bloodstains, and hair forensic samples from California. Calif. Fish Game.

[83] Cairns SJ, Schwager SJ (1987). A comparison of association indices. Anim. Behav..

[84] Godde S, Humbert L, Côté SD, Réale D, Whitehead H (2013). Correcting for the impact of gregariousness in social network analyses. Anim. Behav..

[85] Calenge C (2006). The package ‘adehabitat’ for the R software: A tool for the analysis of space and habitat use by animals. Ecol. Modell..

[86] Team, R. C. R: A language and environment for statistical computing. R Foundation for Statistical Computing, Vienna, Austria. 2013 (2014).

[87] Fieberg J, Kochanny CO (2005). Quanitfying home-range overlap: the importance of the utilization distribution. J. Wildl. Manage..

[88] Whitehead H (2009). SOCPROG programs: Analysing animal social structures. Behav. Ecol. Sociobiol..

[89] Bejder L, Fletcher D, Brager S (1998). A method for testing association patterns of social animals. Anim. Behav..

[90] Whitehead H (1999). Testing association patterns of social animals. Anim. Behav..

[91] Newman MEJ, Girvan M (2004). Finding and evaluating community structure in networks. Phys. Rev. E.

[92] Newman MEJ (2004). Analysis of weighted networks. Phys. Rev. E.

[93] Newman MEJ (2006). Modularity and community structure in networks. Proc. Natl. Acad. Sci. USA.

[94] Borgatti, S. NetDraw Software for Network Visualization (2002).

[95] Wang J (2007). Triadic IBD coefficients and applications to estimating pairwise relatedness. Genet. Res..

[96] Wang J (2011). COANCESTRY: A program for simulating, estimating and analysing relatedness and inbreeding coefficients. Mol. Ecol. Resour..

[97] Duchesne P, Étienne C, Bernatchez L (2006). PERM: a computer program to detect structuring factors in social units. Mol. Ecol. Notes.

